# Iws1 and Spt6 Regulate Trimethylation of Histone H3 on Lysine 36 through Akt Signaling and are Essential for Mouse Embryonic Genome Activation

**DOI:** 10.1038/s41598-019-40358-3

**Published:** 2019-03-07

**Authors:** Reza K. Oqani, Tao Lin, Jae Eun Lee, Jeong Won Kang, Hyun Young Shin, Dong Il Jin

**Affiliations:** Department of Animal Science & Biotechnology, Research Center for Transgenic Cloned Pigs, Chungnam National University, Daejeon, 34134 Republic of Korea

## Abstract

The mRNA processing and export factor, Iws1, interacts with the histone H3/H4 chaperone, Spt6 (Supt6 in mouse gene ontology) and recruits the lysine methyltransferase, Setd2, to chromatin to regulate H3K36me3. This recruitment is known to be crucial for pre-mRNA splicing and Iws1 has been shown to interact with REF1/Aly to mediate mRNA export. However, the role of this complex has not yet been examined in embryonic development. Here, we show that knockdown of either *Iws1* or *Supt6* blocked embryo development, primarily at the 8/16-cell stage, indicating that Iws1 and Supt6 are crucial for mouse preimplantation development. In the knockdown embryos, we observed decreases in pre-mRNA splicing, mRNA export and the expression of the lineage-specific transcription factor, Nanog. We found that either Iws1 or Supt6 are required for H3K36 trimethylation and that concurrent knockdown of both *Iws1* and *Supt6* blocks embryonic development at the 2-cell stage. We show that H3K36me3 is modulated by the Pi3k/Akt pathway, as inhibition of this pathway reduced the global level of H3K36me3 while activation of the pathway increased the level of this modification in 2-cell embryos. We observed that Iws1 interacts with nuclear Akt in early embryos, and herein propose that Akt modulates H3K36me3 through interaction with Iws1. Together, our results indicate that the Iws1 and Supt6 play crucial roles in embryonic genome activation, lineage specification, and histone modification during mouse early development.

## Introduction

Embryonic genome activation (EGA) is the highly complex process through which the transcriptionally silenced embryo produced via fertilization of two highly differentiated gametes is reprogrammed into a totipotent embryo with its own transcriptional activity. This process is governed by the regulation of gene expression at all levels, including but not limited to mRNA transcription and post-transcriptional events^1,2^.

Reprogramming is mainly a result of changes in the access of RNA Polymerase II (Pol II) to gene promoters and the availability of transcription factors. Although transcription factors play major roles in triggering developmental gene expression, additional factors regulate the promoter access of Pol II by altering specific chromatin states throughout development^3^.

It has been well established that there is a complex relationship among transcription, post-transcriptional processing, and chromatin structure^4^. One molecular mechanism known to regulate chromatin structure and gene expression is the chemical modification of histone proteins, various amino acids of which can be modified by phosphorylation, acetylation, methylation or ubiquitination. For example, the acetylation of histone lysine is usually linked to gene activation, whereas lysine methylation can correlate with gene repression or activation depending on which residues and histones are modified^5^.

Histone H3 can be mono-, di, or tri-methylated on lysine 36 and these methylations are abundant and highly conserved histone modification in eukaryotes. In yeast, all H3K36 methylations exist and are generated by the single histone methyltransferase, Set2^6^. In yeast, it has been well established that the tri-methylation of H3K36 directs the deacetylation of histones behind the transcribing Pol II, thereby suppressing the initiation of intragenic transcription^7–9^. This maintains the accuracy of Pol II transcription by suppressing the incorporation of acetylated histones and signaling for these histones to be deacetylated in transcribed genes^10^.

The activity of elongation factors is required for proper K36 trimethylation. In yeast, the essential elongation factor complex, Bur1/Bur2, which includes the catalytic and regulatory subunits of a cyclin-dependent kinase, is necessary for Pol II CTD Ser2 phosphorylation and transcription elongation^11–13^. Bur1 kinase activity has been shown to be crucial for K36 trimethylation, along with Set2 methyltransferase activity^14,15^. The Bur kinase substrate, Spt5, is an essential yeast Pol II elongation factor and is also necessary for K36 trimethylation^15^.

In human, KMT3A (SETD2) is responsible for virtually all global and transcription-dependent H3K36me3, but not -me1 or -me2^16^. The mono- and dimethylations of H3K36 are regulated by other histone methyltransferases, including NSD1, which adds both mono- and dimethylation to this residue^17^.

Similar to yeast Set2, human SETD2 is associated with the elongation phase of Pol II transcription and physically binds to hyper-phosphorylated Pol II^18,19^. Furthermore, studies have shown that SETD2 is recruited to the Pol II elongation complex via an interaction with the CTD-bound elongation factor complex, Spt6:Iws1 and that depletion of Iws1 abrogates the level of elongation-coupled H3K36me3^20^.

Spt6 (Supt6 in mouse) is an H3/H4 histone chaperone that binds to Pol II CTD specifically when the CTD is phosphorylated at Ser2 (not Ser5 or Tyr1)^21^. Depletion of Spt6 or a Spt6 mutation that disrupt the Spt6-Pol II CTD interaction leads to defects in pre-mRNA splicing and mRNA export in human cells^21,22^.

Iws1 is a Pol II elongation factor that was originally discovered in yeast *S*. *cerevisiae* as an essential protein that interacts with Spt6^23^. Human IWS1 interacts with the Spt6 homolog, SUPT6h^21^, and is essential for cell viability^24^. IWS1 reportedly interacts with the nuclear mRNA export factor, REF1/ALY, and depletion of IWS1 results in the nuclear retention of bulk mRNA in HeLa cells^21^. Thus, it appears that the interaction of SUPT6h with Pol II CTD Ser2p mediates the co-transcriptional recruitment of IWS1, REF1/ALY, and various mRNA processing and export factors to their responsive genes^21^.

Despite the importance of Iws1 and Supt6 in regulating gene expression, however, no previous study has examined their presence and function in embryonic development. Here, we report that Iws1 and its interacting partner, Supt6, are important in mouse early development. We show that these factors are present in oocytes and embryos and that they are crucial for proper preimplantation development, global gene expression and lineage factor regulation in mouse embryos. In addition, we reveal that simultaneous depletion of Iws1 and Supt6 blocks mouse embryo development beyond EGA.

## Results

### Supt6 and Iws1 are expressed and interact in mouse oocytes and early embryos

**Figure 1:**
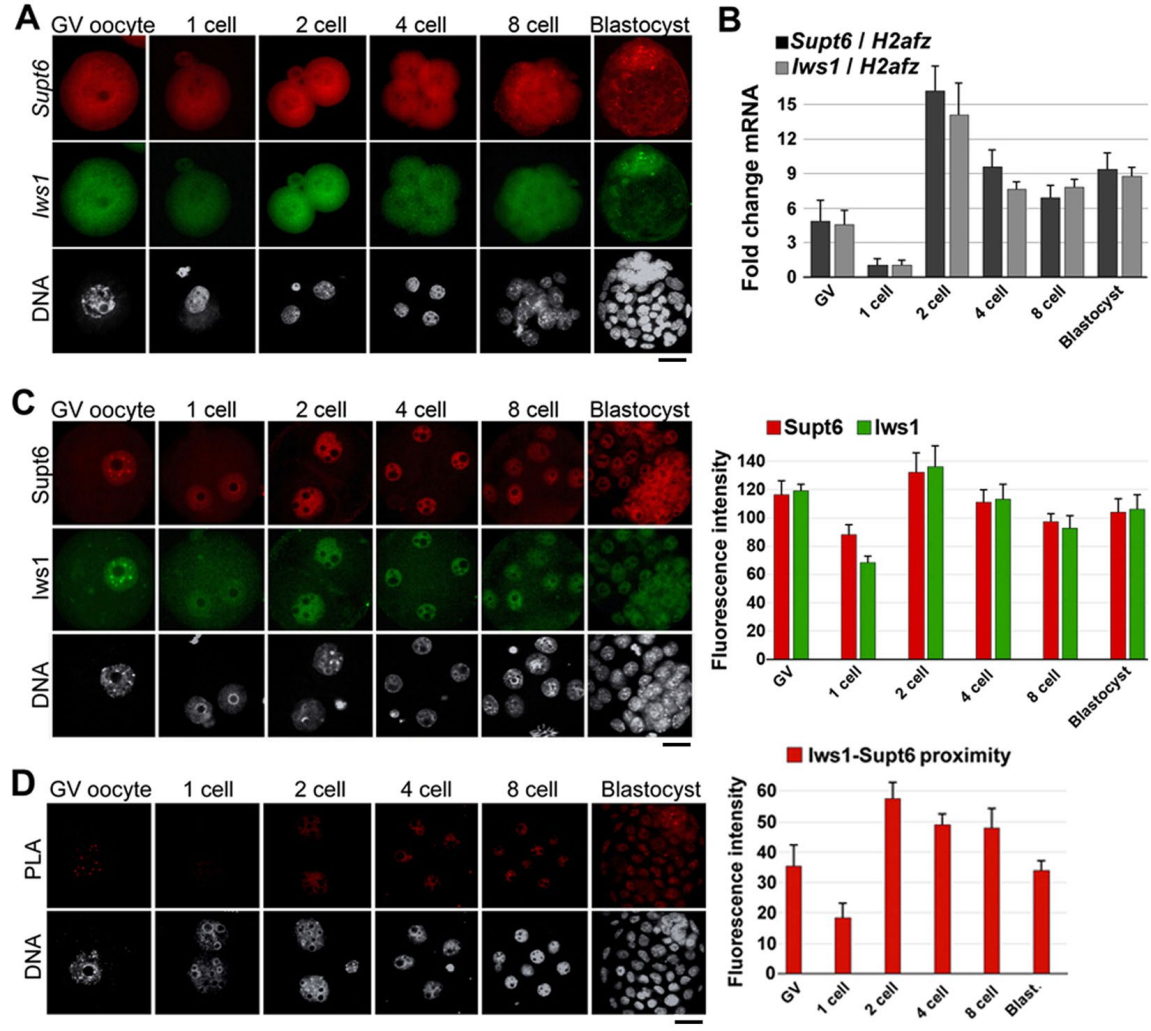
Expression and localization of Supt6 and Iws1 in mouse early development. (**A**) FISH analysis of *Supt6* and *Iws1* mRNA expression in GV oocytes and preimplantation embryos. (**B**) The RT-qPCR analysis shows the dynamic expression of both *Supt6* and *Iws1* mRNAs in mouse early development. The expression level of *H2afz* was detected as an internal control. The mRNA level observed in 1-cell embryos was defined as 1. Fifty embryos from each stage were used for analysis. (**C**) Nuclear localization of Supt6 and Iws1 proteins were analyzed by double immunostaining in GV oocytes and post-fertilization embryos. At least 15 oocytes or embryos of each stage were analyzed by confocal microscopy, and the fluorescence intensities of nuclear signals were measured. The mean intensity of each analysis is depicted in the graphs. (**D**) The Iws1-Supt6 interaction was examined by proximity ligation assay (PLA). Fluorescence signals were detected by confocal microscopy; the intensities of nuclear signals were measured and are depicted in the graph. DNA was counterstained with DAPI. Scale bars: 20 μm.

To gain insight into the function of Iws1:Supt6 in mouse early development, we examined the expression of *Iws1* and *Supt6* and the localization of Iws1 and Supt6 in oocytes and preimplantation embryos. FISH analysis of *Iws1* and *Supt6* revealed that both mRNAs were present at all examined stages, mainly in the cytoplasm [Fig. 1A]. RT-qPCR analysis of *Iws1* and *Supt6* mRNA expression in preimplantation embryos revealed that the expression levels of these mRNAs peaked at the 2-cell stage and gradually decreased at 4-cell stage and then slightly increased at 8-cell stage and thereafter [Fig. 1B].

Next, we used immunocytochemistry to analyze the protein localization of Iws1 and Supt6 in oocytes and embryos. Double immunostaining revealed that the proteins showed nuclear colocalization in oocytes and embryos at all analyzed stages [Fig. 1C]. Clear colocalization of Iws1 and Supt6 was observed in the nuclei of GV oocytes, suggesting that these proteins are maternally contributed to early development. The nuclear accumulation of Iws1 and Supt6 continued throughout the preimplantation stages; the highest level was seen at the 2-cell stage, and the fluorescence intensity was relatively consistent thereafter. To examine whether Iws1 and Supt6 might physically interact, we used proximity ligation assays (PLA) to determine whether these proteins reside in close proximity (less than 40 nm) to one another, and therefore have the potential to interact^25,26^. We observed that Iws1 and Supt6 were in close proximity at all examined stages and that this proximity was substantially higher at the 2-cell stage than at the other stages [Fig. 1E].

### Depletion of either *Iws1* or *Supt6* impairs early embryonic development

**Figure 2:**
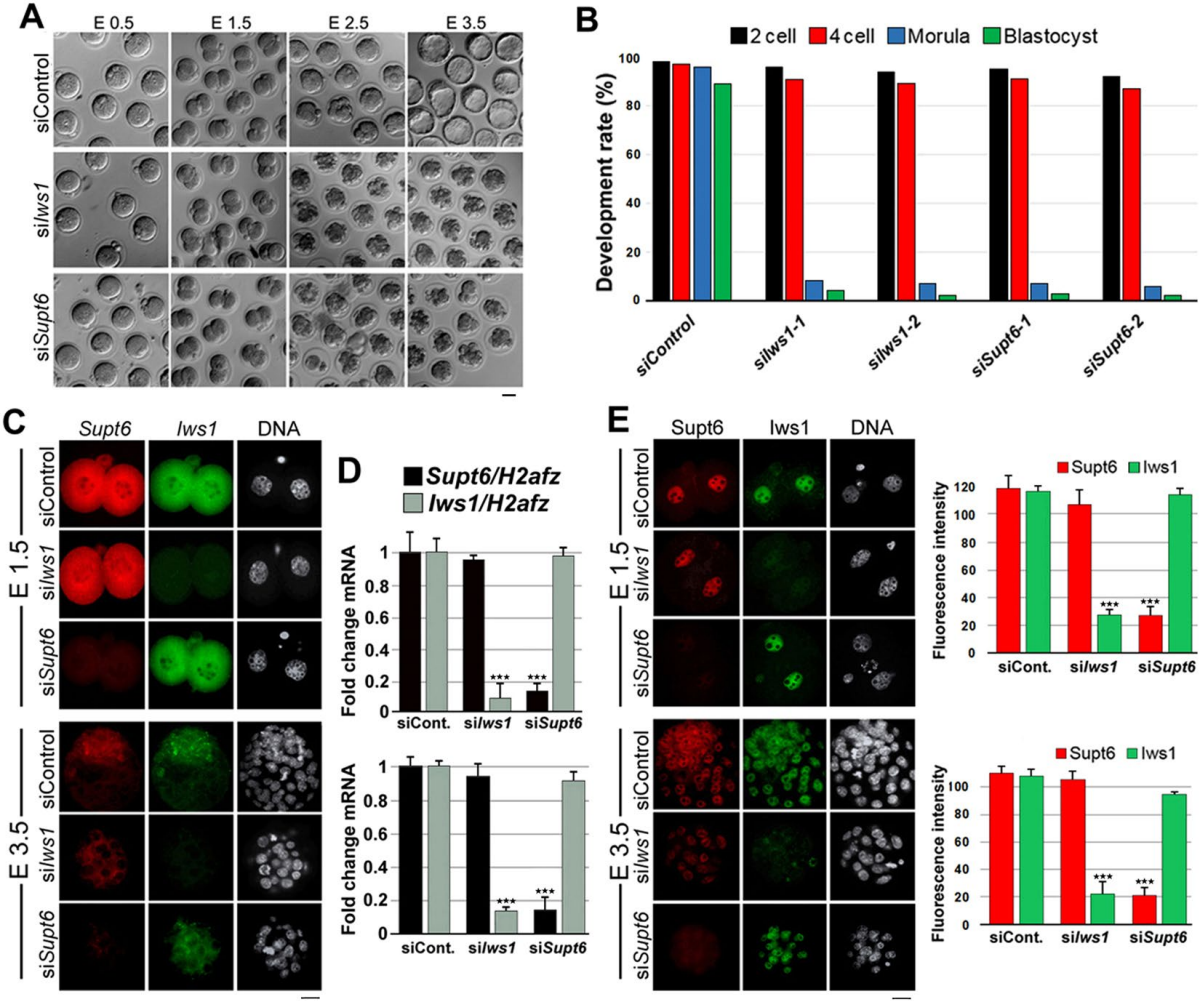
Effects of *Iws1*- or *Supt6* knockdown on embryo development. (**A**) Representative images showing the developmental consequences of electroporating control siRNA (siControl), *Iws1* siRNA (si*Iws1*) or *Supt6* siRNA (si*Supt6*) into mouse early zygotes. Scale bar: 50 μm. Photographs were taken at the indicated embryonic days. (**B**) The graph depicts the percentage of development rates in control and knockdown embryos. (**C**) si*Iws1* reduces the mRNA level of *Iws1* but not *Supt6*, while the reverse is true for si*ISupt6* in mouse embryos at E1.5 and E3.5. Zygotes were electroporated with siControl, si*Iws1* or si*Supt6* and FISH was used to simultaneously label *Iws1* mRNA and *Supt6* mRNA in embryos at 28 hr and 80 hr after electroporation (44 and 96 hphCG, respectively). Scale bar: 20 μm. (**D**) The RT-qPCR analysis confirms that *Iws1* mRNA and *Supt6* mRNA are efficiently and specifically depleted in si*Iws1* and si*Supt6* embryos, respectively, at E1.5 and E3.5. (**E**) In mouse embryos at E1.5 and E3.5, si*Iws1* reduces the nuclear levels of Iws1 but not Supt6, while si*Supt6* has the opposite effect. Zygotes were electroporated with siRNA, and Iws1 and Supt6 proteins were co-immunostained in embryos at 28 hr and 80 hr after electroporation (44 and 96 hphCG, respectively). DNA was counterstained with DAPI. The fluorescence intensities corresponding to each labeled protein were measured in at least 20 embryos per group. The mean intensity of each group is depicted in the graphs corresponding to each embryonic day. Scale bar: 20 μm.

Interestingly, when the zygotes were electroporated with *Iws1* siRNA or *Supt6* siRNA at ~16 hours post hCG injection (hphCG, embryonic day E 0.5) and cultured for an additional 80 hr, the majority of the embryos failed to progress to the morula stage [Fig. 2A]. In fact, almost all knockdown embryos arrested at the 8/16-cell stage, whereas zygotes electroporated with control siRNA developed to blastocysts. To confirm that the observed developmental arrest was due to the introduced *Iws1* siRNA or *Supt6* siRNA, we used a second siRNA approach in which we introduced pools of three different siRNA, all targeting the same mRNA. We obtained the same result with the second set of siRNA [Fig. 2B]. To confirm the siRNA specificity in our *Iws1*- and *Supt6*-knockdown embryos, we used FISH, RT-qPCR, and immunostaining. In *Iws1*-knockdown embryos, FISH revealed a dramatic reduction of *Iws1* mRNA signal intensity with no significant change in *Supt6* mRNA intensity in embryos examined at embryonic day (E)1.5 and E3.5 [Fig. 2C]. The reverse was seen for *Supt6*-knockdown embryos [Fig. 2C]. The RT-qPCR analysis confirmed that the mRNA expression levels of *Iws1* and *Supt6* were dramatically reduced in siIws1 and siSupt6 embryos, respectively, at both E1.5 and E3.5 [Fig. 2D]. Finally, immunohistochemistry revealed that *Iws1* knockdown significantly reduced the nuclear level of Iws1 but not that of Supt6 in embryos examined at E1.5 and E3.5 [Fig. 2E]. Together, these results show that Iws1 and Supt6 are crucial factors for mouse early embryogenesis.

### Knockdown of *Iws1* or *Supt6* induces defects in mRNA splicing and export

**Figure 3:**
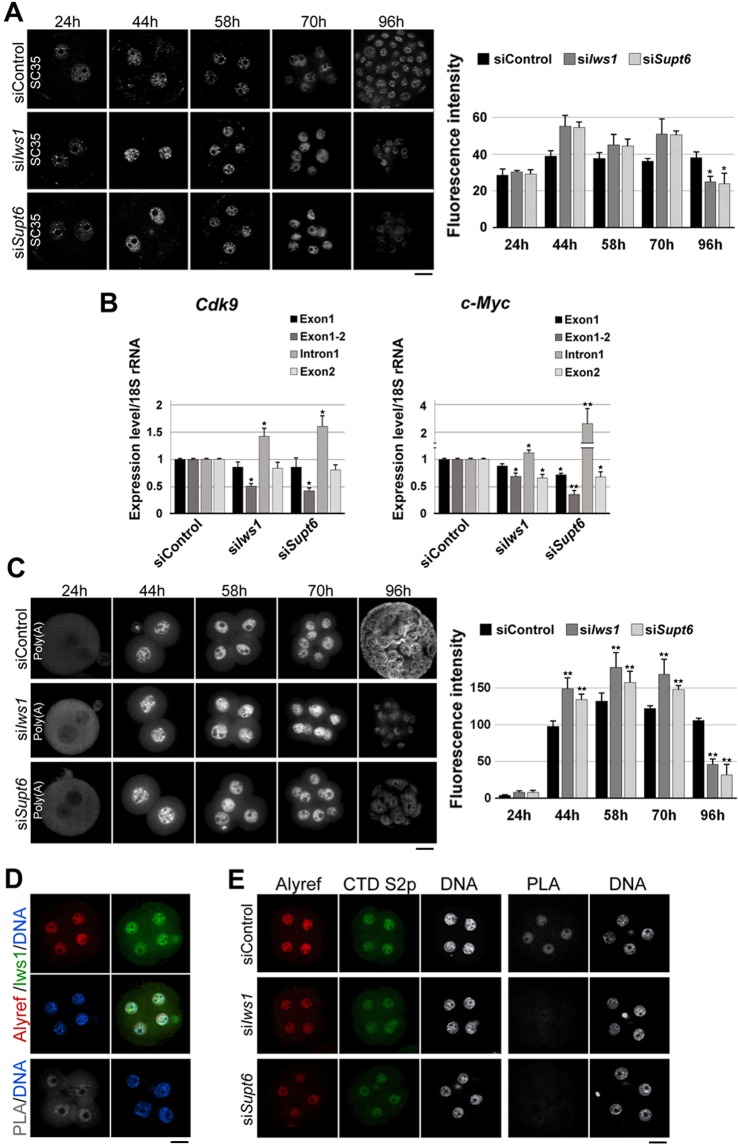
Effects of si*Iws1* or si*Supt6* on mRNA splicing and export in mouse embryos. (**A**) Zygotes were electroporated with siControl, si*Iws1* or si*Supt6* and cultured to the blastocyst stage. At the indicated times, splicing speckles were detected using anti-SC35. At least 15 embryos were used for each group. The fluorescence intensity of SC35 was measured in each specimen; mean values are depicted in the graph. (**B**) The pre-mRNA splicings of *Cdk9* and *c-Myc* are defective in si*Iws1* and si*Supt6* embryos. The levels of spliced and unspliced intron 1 were quantified by RT-qPCR. The expression level of the 18 S rRNA in siControl was set as 1 and used as an internal control. (**C**) Bulk Poly(A)+ mRNA accumulation in si*Iws1* or si*Supt6* embryos. Poly (A)+ mRNAs were labeled by FISH using a specific oligo-dT probe and analyzed by confocal microscopy. The fluorescence intensities are depicted in the graph. At least 15 embryos were analyzed for each stage. (**D**) Iws1 colocalizes and interacts with Alyref. Four-cell embryos were double-immunostained against Iws1 and Alyref. PLA shows the putative interaction of the two factors. DNA was counterstained with DAPI. (**E**) Four-cell stage siControl, si*Iws1*, and si*Supt6* embryos were double-stained for Alyref and Pol II CTD Ser2p. PLA shows that either Iws1 or Supr6 is required for the interaction of Alyref with Pol II CTD Ser2p. Scale bars: 20 μm.

Studies have shown that IWS1 and SUPT6h contribute to pre-mRNA splicing by recruiting SETD2 to chromatin^20^. To investigate whether these factors are involved in the pre-mRNA splicing of mouse early embryos, we first analyzed the effect of *Iws1* or *Supt6* depletion on the distribution of splicing speckles throughout development. Immunostaining for the splicing speckle marker, SC35, revealed that splicing speckles were more pronounced and accumulated, specifically at the 2-cell stage, in knockdown (KD) embryos compared to siControl embryos [Fig. 3A]. The fluorescence intensity of the SC35 nuclear signal was significantly reduced at 96 hphCG (E3.5) in KD embryos compared to their siControl counterparts.

Next, we used RT-qPCR analysis to specifically examine the splicing of *Cdk9* and *c-Myc* pre-mRNAs. We found that first introns of *c-Myc* pre-mRNA and *Cdk9* pre-mRNA were efficiently spliced (removed) in siControl embryos, whereas KD embryos exhibited dramatic accumulations of these introns [Fig. 3B]. Thus, there appear to be a pre-mRNA splicing defects in KD embryos.

IWS1 reportedly interacts with the nuclear export factor, REF1/ALY, and *IWS1-*knockdown HeLa cells exhibited disruption of nuclear mRNA export^20,21^. To test whether Iws1:Supt6 has a similar function in mouse early embryos, we used FISH to label poly(A)+ mRNAs in siControl, si*Iws1* and si*Supt6* embryos at various stages. Confocal microscopy revealed that there was no detectable signal in the pronuclei of the examined zygotes [Fig. 3C]. A clear nuclear signal was detected in siControl embryos at the 2-cell stage (44 hphCG, E 1.5); this nuclear signal was increased at the 4-cell stage, gradually decreased to the 8-cell stage and became nucleo-cytoplasmic in blastocysts (96 hphCG, E3.5). The accumulation of poly(A)+ mRNA was higher in the nuclei of 2-cell, 4-cell and 8-cell si*Iws1* embryos compared to their siControl counterparts, but the intensity of the nuclear signals was drastically lower in 8/16-cell-arrested si*Iws1* embryos (96 hphCG, E3.5). Similar changes in the abundance of poly(A)+ mRNA were observed in si*Supt6* embryos. Thus, our results indicate that Iws1 and Supt6 are separately necessary for proper mRNA nuclear export during mouse early embryogenesis.

To further investigate whether Iws1:Supt6 is directly involved in mRNA nuclear export, we examined the possible interaction of Iws1 and nuclear mRNA export factor, Alyref in mouse early embryos at the 4-cell stage (58 hphCG), when the nuclear intensity of poly(A)+ mRNA was maximized. Double immunostaining revealed that Alyref localized to the cell nuclei of embryos, where it colocalized with Iws1. PLA experiment also confirmed the close proximity of these factors [Fig. 3D], indicating that they may interact with each other in mouse embryos. We also observed that the proximity of Alyref and Ser2p was significantly decreased in knockdown embryos compared to siControl embryos [Fig. 3E]. This is the first report to show that Iws1 may interact with Alyref in murine cells. Moreover, our data show that Iws1:Supt6 is important for the interaction of Alyref and Pol II CTD for proper mRNA nuclear export in mouse early embryos.

### Iws1 is necessary for the regulation of Nanog

**Figure 4:**
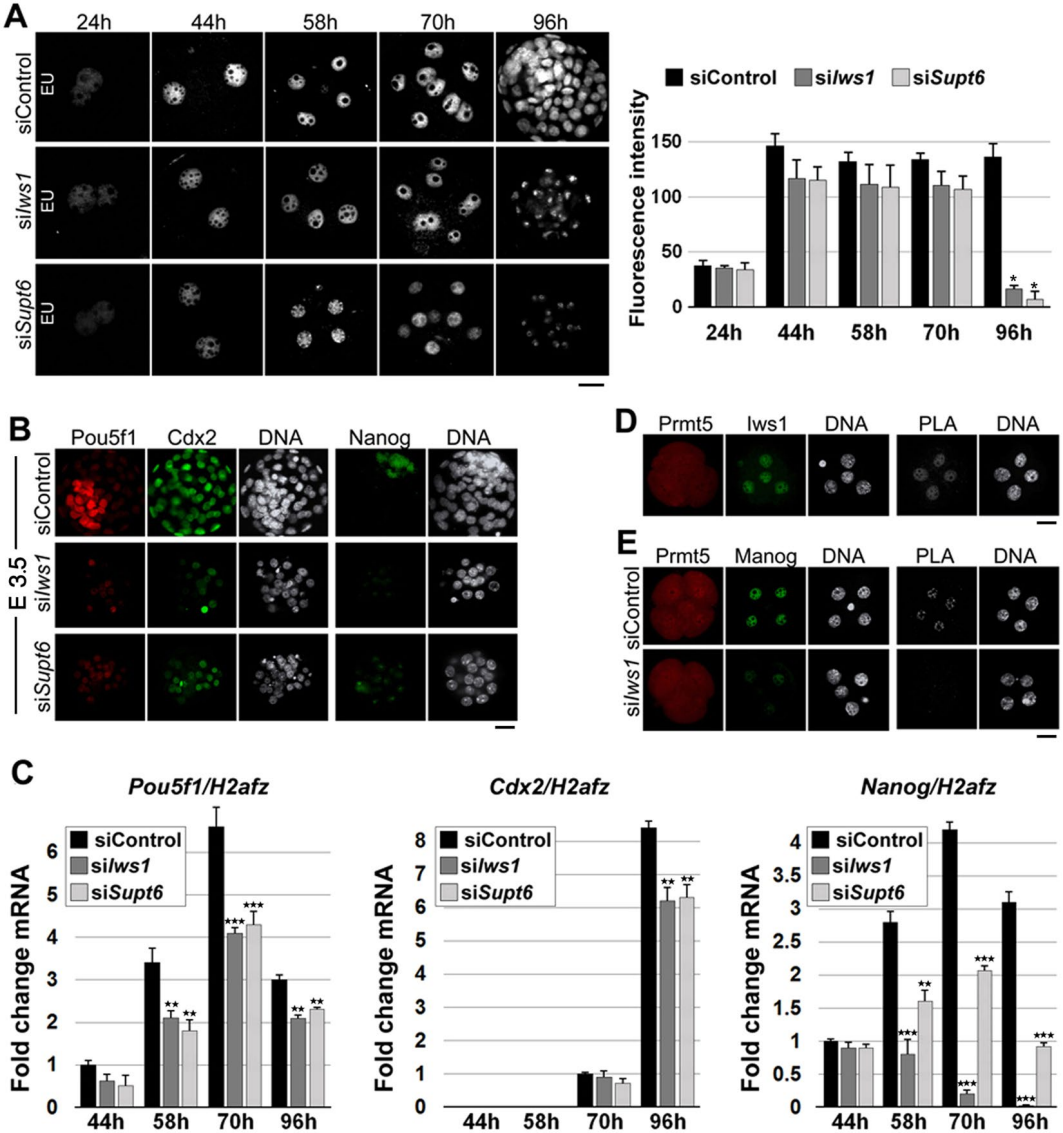
Iws1 is required for the expression of Nanog. (**A**) EU-derived fluorescence was used to assess the global transcription in embryos electroporated with the indicated siRNAs. The fluorescence intensity of EU is highest in 2-cell stage siControl embryos and exhibits drastic decreases in si*Iws1* and si*Supt6* embryos at 96 hphCG. At least 20 embryos were used for each experiment. (**B**) Immunofluorescent detection of Pou5f1 (Oct4), Cdx2 and Nanog in siControl, si*Iws1* and si*Supt6* embryos. Representative images of E3.5 embryos are shown. DNA was counterstained with DAPI. At least 30 embryos were used for each experiment. (**C**) RT-qPCR analysis of the *Pou5f1*, *Cdx2* and *Nanog* mRNA in siControl, si*Iws1* and si*Supt6* embryos at the indicated times after hCG injection. The mRNA expression of *H2afz* was used as an internal control. The mRNA expression levels of *Pou5f1*, *Cdx2* and *Nanog* in siControl embryos at 44 hr, 70 hr, and 44 hr, respectively, were defined as 1. One-hundred embryos were used for each analysis. (**D**) PLA showing the apparent interaction of Prmt5 and Iws1 in the cell nuclei of representative 4-cell stage embryos. (**E**) PLA showing that Prmt5 and Nanog interact in early embryos and that Iws1 is required for this interaction. DNA was counterstained with DAPI. At least 20 embryos were used for each experiment. Scale bars: 20 μm.

To elucidate the effect of *Iws1* or *Supt6* KD on embryonic gene expression, we used 5-Ethinyl Uridine (EU) incorporation assays to analyze the global transcription in control and KD embryos. In si*Iws1* and si*Supt6* embryos, the nascent RNA levels were only slightly lower than those in siControl embryos up to the 8-cell stage (70 hphCG, E2.5). At 96 hphCG (E3.5), however, the level of EU labeling was drastically reduced in KD embryos compared to siControl embryos [Fig. 4A]. We also observed that the nuclear intensities of the pluripotency factors, Pou5f1 (Oct4) and Nanog, were notably decreased in KD embryos relative to siControl embryos [Fig. 4B]. RT-qPCR analysis showed that in siControl embryos, the *Pou5f1* mRNA level increased after 44 hphCG (2-cell stage), peaked at 70 hphCG (8-cell stage) and decreased at 96 hphCG (blastocyst stage), while the *Pou5f1* mRNA level in KD embryos showed a similar pattern but was significantly lower than that of siControl group at all analyzed stages [Fig. 4C]. *Cdx2* mRNA expression was first detected at 70 hphCG and increased at 96 hphCG in siControl embryos; in KD embryos, it was slightly reduced at 70 hphCG and significantly reduced at 96 hphCG, relative to controls [Fig. 4C]. Interestingly, the mRNA expression of *Nanog* was dramatically decreased in KD embryos relative to siControl embryos, particularly in si*Iws1* embryos. In siControl embryos, *Nanog* mRNA expression increased gradually, peaked at 70 hphCG and decreased at 96 hphCG. In KD embryos, in contrast, *Nanog* mRNA expression was severely suppressed starting at 70 hphCG. Indeed, no *Nanog* expression was detectable in si*Iws1* embryos at 96 hphCG [Fig. 4C]. These observations prompted us to speculate that there could be a relationship between Iws1 and Nanog in early mouse embryos. IWS1 is known to physically interact with PRMT5 in human cells^24^ and Prmt5 was shown to interact with Nanog in mouse embryonic stem cells^27^. Indeed, PLA revealed that Iws1 and Prmt5 exist in close proximity and thus may interact with each other in the nuclei of mouse blastomeres [Fig. 4D]. We also found that Prmt5 and Nanog exist in close proximity and thus may interact in the nuclei of mouse blastomeres, and this interaction was disrupted by *Iws1* knockdown [Fig. 4E]. Based on these findings, we conclude that Iws1 regulates Nanog expression through interaction with Prmt5.

### Either Iws1 or Supt6 is necessary for H3K36 trimethylation

**Figure 5:**
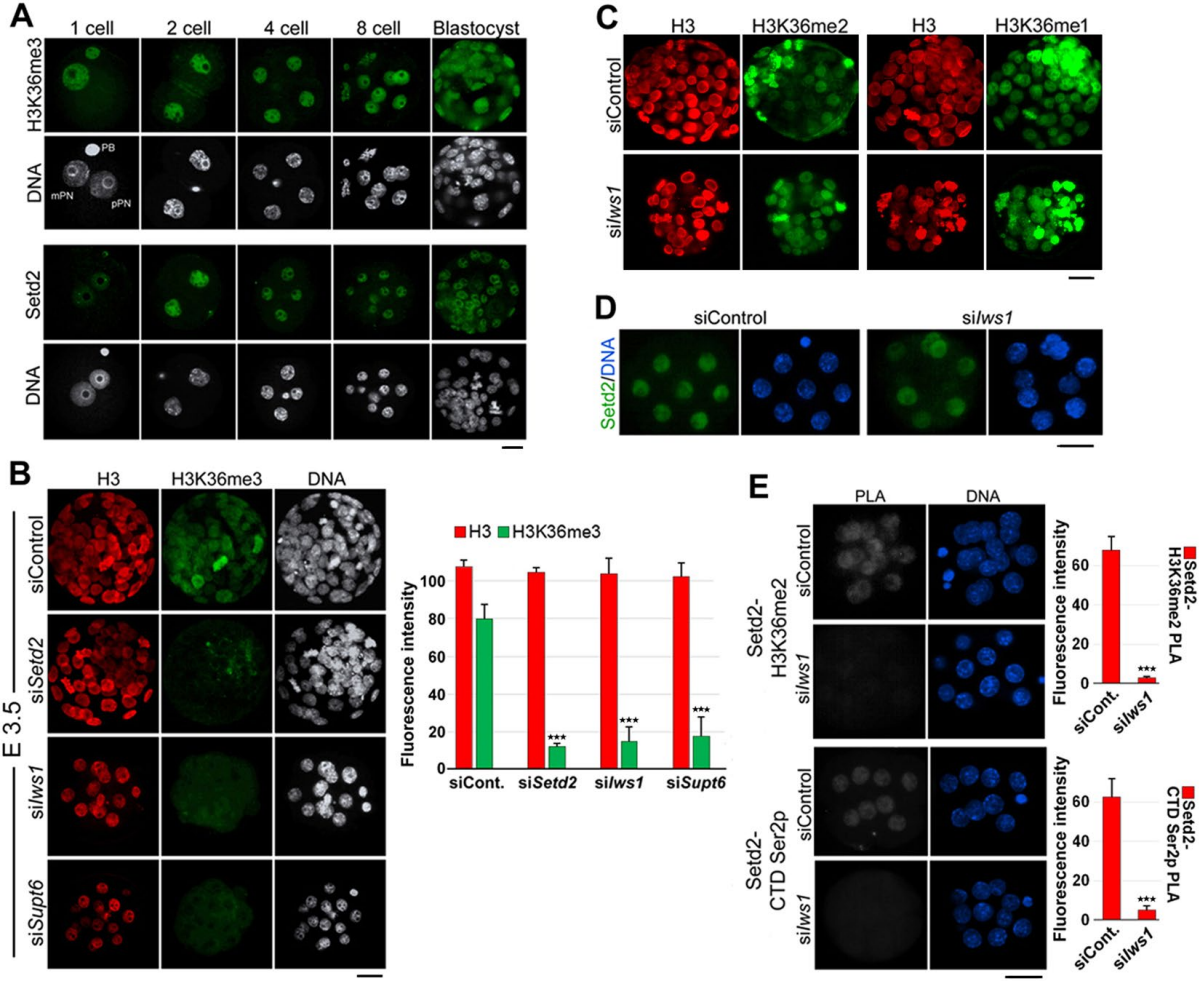
Iws1 is specifically required for H3K36 trimethylation in mouse preimplantation embryos. (**A**) The Immunocytochemical analysis shows that H3K36me3 and Setd2 are present during preimplantation development. With the exception of the zygote stage, in which H3K36me3 appears only in the maternal genome, this modification appears on chromatin during all analyzed stages. Setd2 is present at all stages of preimplantation development. (**B**) Knockdown of *Setd2*, *Iws1* or *Supt6* dramatically reduces the level of H3K36me3 compared to that in the siControl group but does not affect the level of H3. DNA was counterstained with DAPI. (**C**) *Iws1* knockdown does not affect H3K36me2 or -me1 at E3.5 (80 hr after siRNA introduction). (**D**) Immunofluorescence staining shows that *Iws1* knockdown does not significantly affect the nuclear level of Setd2. (**E**) PLA shows that Iws1 is necessary for the interactions of Setd2 with H3K36me2 and Pol II CTD Ser2p. The fluorescence intensities of the PLA signals were dramatically reduced in si*Iws1* embryos compared to siControl embryos. At least 20 embryos were used for each experiment. Scale bars: 20 μm.

H3K36 methylations have been linked to the transcriptionally active chromatin state; among them, K36me3 has been linked directly to Setd2 methyltransferase activity^16^. As Iws1:Supt6 is reportedly required for Setd2-mediated K36 trimethylation in yeast and human^20,28,29^, we examined whether these factors are important for the establishment of K36me3 in mouse early embryos. We first examined the presence and kinetics of this histone mark in mouse early embryos [Fig. 5A]. In zygotes, immunostaining revealed that the HK36me3 signal was limited to the maternal genome, with the male pronucleus showing an almost complete lack of staining. At the 2-cell stage and onward, K36me3 was observed throughout development at interphase chromatin and all mitotic chromosomes of all examined stages. Setd2 was also present during all examined stages; it showed nuclear localization from the zygote to blastocyst stage, with the lowest intensity seen in zygotes and the highest intensity seen in 2-cell embryos [Fig. 5A].

To confirm that the utilized anti-H3K36me3 antibody bound specifically to its target antigen, we exposed mouse zygotes to si*Setd2* or siControl and compared them by immunostaining at 96 hphCG (E3.5). Indeed, si*Setd2* embryos showed a dramatic decrease in the H3K3me3 signal without any significant change in the level of H3 [Fig. 5B], while H3K36me1 or H3K36me2 showed almost no change in these embryos [Fig. S1], confirming that the signal from H3K36me3 labeling was genuine. When si*Iws1* or si*Supt6* was applied, embryo development stopped at the 8/16 cell stage, as expected. We then performed double immunostaining of embryos at 96 hphCG, in an effort to understand whether any change in H3K36me3 level was due to a change in H3 level. No nuclear signal from K3me3 was observed in these embryos, whereas the nuclear intensity of H3 remained relatively unchanged [Fig. 5B]. The fluorescence intensity of both H3 and H3K36me3 was measured in each specimen and summarized in a graph [Fig. 5B].

We next questioned whether *Iws1* knockdown had any effect on other H3K36 methylations. Since the literature lacked any report on H3K36me1 or -me2 in mouse preimplantation embryos, we first examined these two histone marks separately in mouse early embryos. Immunostaining revealed that both histone modifications are present from the zygote to blastocyst stages of mouse embryos [Fig. S2]. When we introduced si*Iws1* into zygotes and observed the embryos at 96 hphCG, we did not observe any significant change in the K36me1 or -me2 signals compared to those in untreated embryos [Fig. 5C].

Given that si*Iws1* significantly reduced the K36me3 signal, we tested whether this effect was due to a down-regulation of Setd2 expression or some change in its interactions. We examined the nuclear intensity of this factor in *Iws1*-knockdown and control embryos at the 8-cell stage but failed to observe any significant change in the nuclear intensity of Setd2 between the two groups [Fig. 5D]. Therefore, we investigated if *Iws1* knockdown disrupted the interactions of Setd2. PLA revealed that, as expected, Setd2 existed in close proximity to Pol II CTD Ser2p and its substrate, H3K36me2, in siControl embryos, but no such proximity was detected in si*Iws1* embryos [Fig. 5E]. These experiments reveal that Iws1 and its partner, Supt6, are crucial regulators of Setd2-mediated H3K36me3 in mouse early embryos.

### Double knockdown of *Iws1* and *Supt6* blocks embryo development at the 2-cell stage

**Figure 6:**
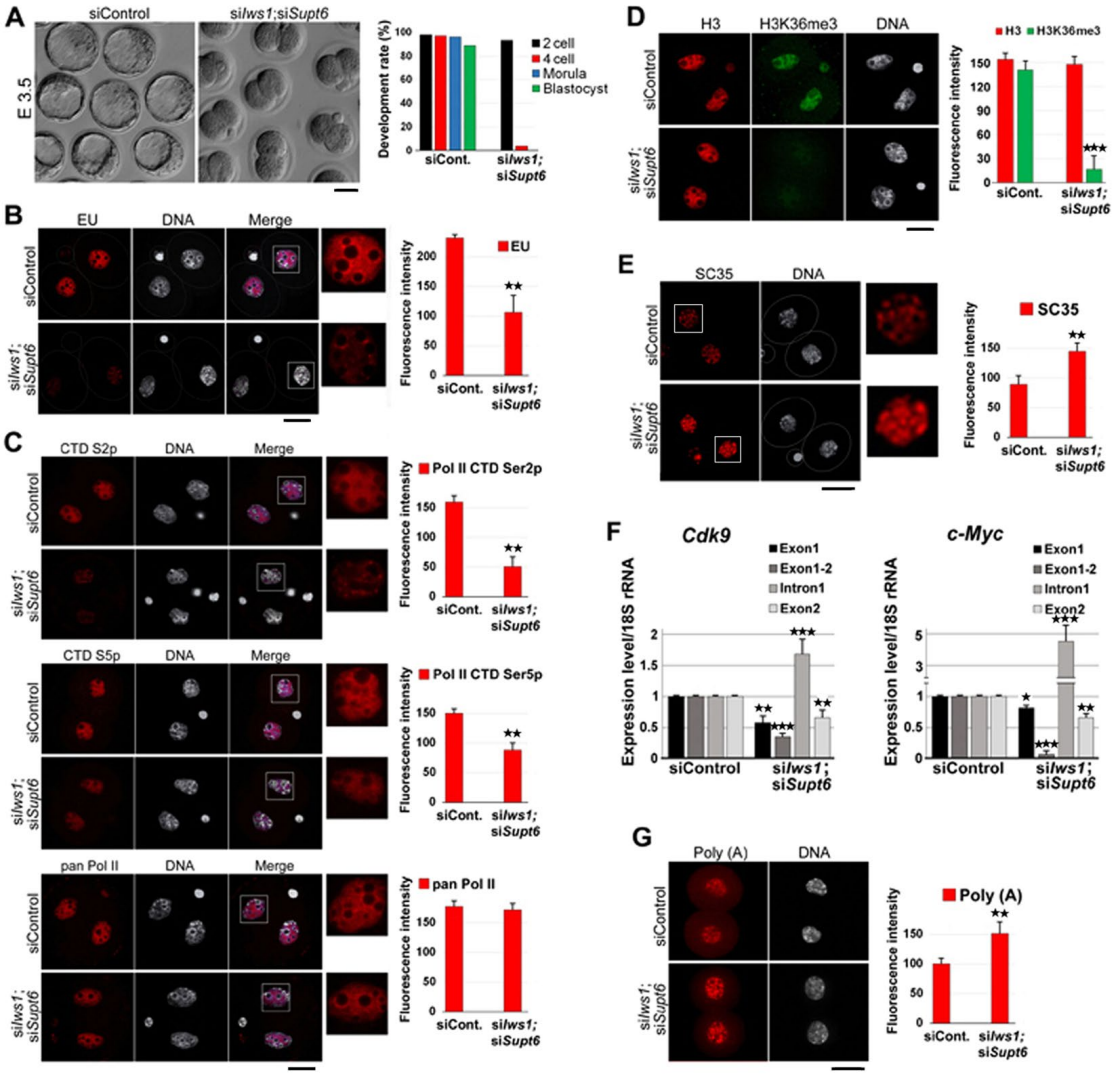
Effects of double knockdown of *Iws1* and *Supt6* on mouse embryo development and global gene expression. (**A**) *Iws1* and *Supt6* double knockdown (si*Iws1*;si*Supt6*) blocks mouse embryo development at the 2-cell stage. More than 95% of the double-knockdown embryos arrest at the 2-cell stage and only 4% reach the 4-cell stage. Scale bar: 50 μm. (**B**) Global nascent RNA transcripts were compared between siControl and si*Iws1*;si*Supt6* 2-cell embryos. The fluorescence intensity of the EU incorporation signal is significantly lower in DKD embryos than in their control counterparts. At least 35 embryos were assessed for each group. (**C**) si*Iws1*;si*Supt6* significantly reduces Pol II CTD Ser2 phosphorylation (CTD S2p) and moderately decreases its Ser5 phosphorylation (CTD S5p) but has little effect on the pan Pol II nuclear signal. In total, 263 embryos were used for the experiment. (**D**) si*Iws1*;si*Supt6* affects H3K36me3 but not H3. The fluorescence intensity of the H3K36me3 nuclear signal is drastically reduced in si*Iws1*;si*Supt6* 2-cell embryos compared to siControl embryos. At least 55 embryos were analyzed in each group. (**E**) *siIws1*;si*Supt6* affects the distribution of the splicing speckle marker, SC35, in 2-cell embryos. Immunofluorescence was used to detect SC35 in siControl and double-knockdown embryos. The fluorescence intensity of the SC35 nuclear signal is significantly higher in si*Iws1*;si*Supt6* 2-cell embryos compared to siControl embryos. DNA was counterstained with DAPI. In total, 65 embryos were analyzed. (**F**) The pre-mRNA splicing of *Cdk9* and *c-Myc* is defective in si*Iws1*;si*Supt6* embryos. The levels of spliced and unspliced intron 1 were quantified by RT-qPCR. The expression level of the 18S rRNA in siControl embryos was set as 1 and used as an internal control. Two hundred embryos were used for each analysis (**G**) si*Iws1*;si*Supt6* induces bulk nuclear poly(A)+ mRNA accumulation in 2-cell embryos. The fluorescence intensity of the poly(A)+ signal is significantly higher in DKD embryos compared to their control counterparts. At least 40 embryos were used for each group. Scale bars: 20 μm.

To explore how the elimination of both *Iws1* and *Supt6* affected early embryo development in the mouse, we used siRNA to simultaneously down-regulate both factors in zygotes. Interestingly, the majority of double knockdown (DKD) embryos failed to progress beyond the 2-cell stage [Fig. 6A]. Also, we observed that transcriptional activity was significantly decreased in DKD embryos compared to control embryos. EU labeling of nascent RNAs at 44 hphCG followed by confocal microscopy revealed that in DKD embryos the nucleoplasmic signal was seriously decreased and nucleolar transcription (surrounding nucleolar precursor bodies) was negatively affected but to a lesser extent [Fig. 6B]. The level of Pol II CTD Ser2p was lower in DKD embryos than in control embryos, whereas the level of Ser5p was only slightly altered and that of pan Pol II level remained relatively unchanged [Fig. 6C]. Immunostaining revealed that the nuclear intensities of Cdk9/Cyclin T1 and Cdk7/Cyclin H were similar in DKD and siControl embryos [Fig. S3]. These data show that Iws1:Supt6 contributes to regulating Pol II-dependent transcription (probably the elongation phase) in mouse early development.

When the levels of pan H3 and H3K36m3 were analyzed by double immunostaining of control and DKD embryos at E1.5 (44 hphCG), we observed a drastic decline in H3K36m3 but no significant change in the level of H3 [Fig. 6D]. As expected, the splicing speckles were redistributed in DKD 2-cell embryo nuclei; moreover, the overall intensity of SC35 was increased in DKD embryos compared to siControl embryos [Fig. 6E]. RT-qPCR-based analysis of the splicing of *Cdk9* and *c-Myc* pre-mRNAs revealed that the first introns of both *c-Myc* pre-mRNA and *Cdk9* pre-mRNA were efficiently spliced in siControl embryos but increasingly retained in DKD embryos [Fig. 6F].

The FISH analysis was used to examine the nuclear abundance of mRNAs in 2-cell embryos. In DKD embryos, the nuclear accumulation of bulk poly(A)+ mRNA and the fluorescence signal intensity were significantly higher in DKD embryos compared to siControl embryos [Fig. 6G]. Together, these findings indicate that mRNA splicing and export are defective in DKD embryos.

### Iws1:Supt6 regulates H3K36me3 through the Akt signaling pathway

As a previous study showed that Iws1 is phosphorylated by Akt3 and Akt1 but not Akt2 in human cells, and these phosphorylations are required for Iws1-mediated Setd2 recruitment^29^, we investigated whether a similar mechanism could govern Iws1 during mouse embryonic development. We first treated mouse 3T3 cells with inhibitors of Akt (MK-2206), Pi3k (LY-294002), Pten (VO-OHpic) or Kdm4 (ML-324) for 6 hr and then subjected the treated cells to double immunostaining against H3 and H3K36me3. This experiment showed that inhibition of Akt or Pi3k reduced H3K36me3 without significantly altering the level of H3, whereas the inhibition of Pten or Kdm4 triggered significant increases in H3K36me3 [Fig. S4]. This confirmed that H3K36me3 is regulated through the Akt signaling pathway in mouse 3T3 cells.

**Figure 7:**
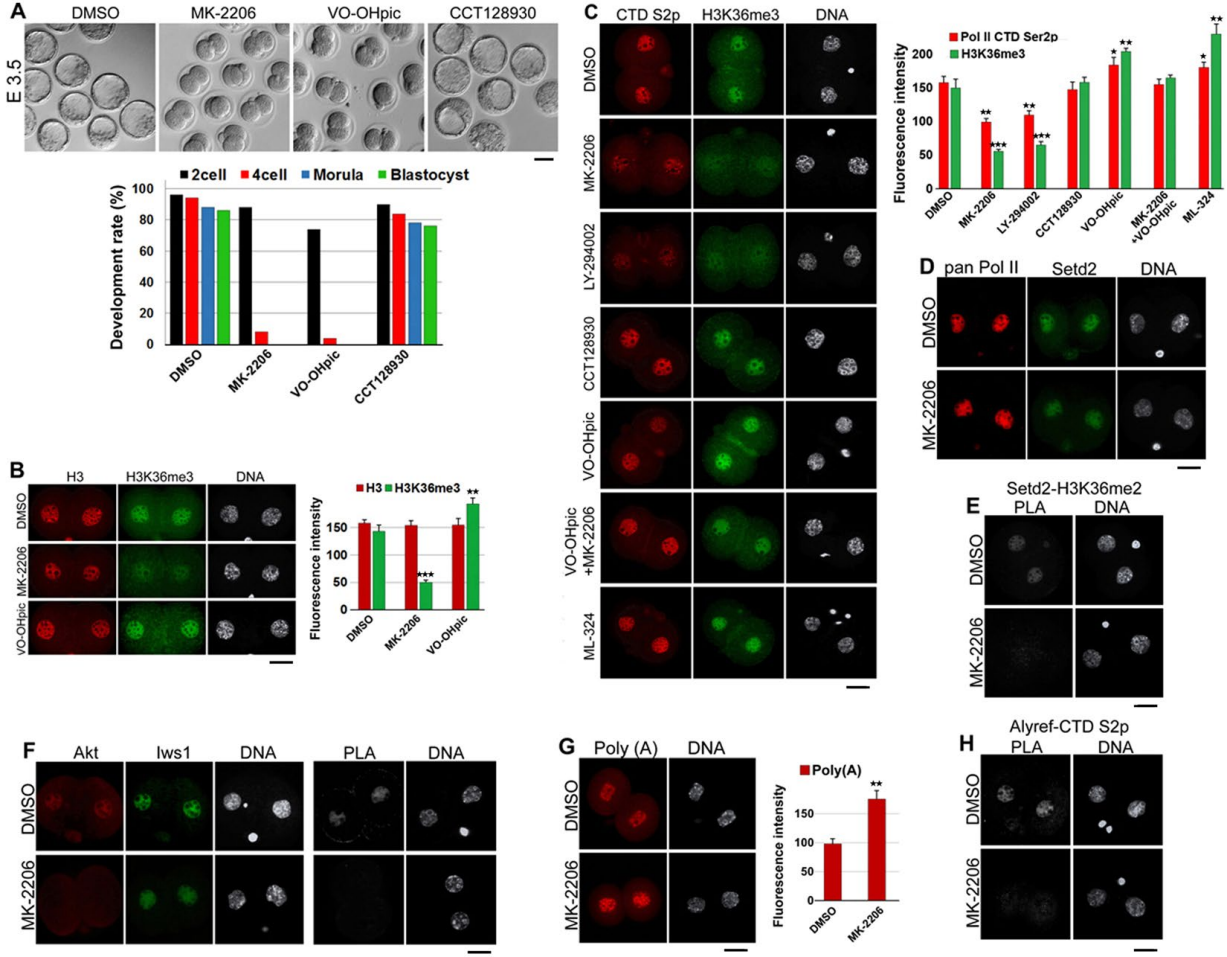
H3K36me3 is regulated through the Pi3k/Akt pathway. (**A**) Treatment with a pan Akt inhibitor (MK-2206) or a Pten inhibitor (VO-OHpic) blocks mouse embryo development at the 2-cell stage, whereas treatment with an Akt2 inhibitor (CCT128930) does not affect blastocyst formation. Scale bar: 50 μm. The graph depicts the development rates of treated embryos. (**B**) Inhibition of pan Akt dramatically decreases the global level of H3K36me3 but not that of H3 in mouse 2-cell embryos. Inhibition of Pten significantly increases the level of H3K36me3 but not that of H3. (**C**) Inhibition of pan Akt or Pi3K by LY-294002, but not Akt2, reduces the levels of both H3K36me3 and Pol II CTD Ser2p, while inhibition of Pten or inhibition of Kdm4 by ML-324 increases the levels of both modifications. Concurrent inhibition of pan Akt and Pten does not significantly affect H3K36me3 or Pol II CTD Ser2p. The graph depicts the mean fluorescence intensities of both modifications in 2-cell embryos after the indicated treatments. In total, 234 embryos were used for this experiment. (**D**) Inhibition of Akt does not significantly affect the nuclear levels of pan Pol II or Setd2. (**E**) PLA shows that pan Akt inhibition disrupts the interaction of Setd2 with H3K36me2. The fluorescence signal is very low in MK-2206-treated embryos compared to their DMSO-treated counterparts. (**F**) Double-immunostaining shows that pan Akt colocalizes with Iws1 in the cell nuclei of 2-cell embryos. Akt inhibition reduces the nuclear signal of Akt but not that of Iws1. PLA reveals that Akt kinase activity is required for the Akt-Iws1 interaction. (**G**) Akt kinase activity is required for proper mRNA nuclear export. MK-2206 induces bulk poly(A)+ accumulation in 2-cell embryos. The fluorescence intensity of nuclear poly(A)+ is significantly higher in MK-2206-treated 2-cell embryos compared to DMSO-treated 2-cell embryos. (**H**) PLA shows that Akt kinase activity is required for the proper interaction of Alyref with Pol II CTD Ser2p (CTD S2p). The fluorescence intensity of the PLA nuclear signal is significantly lower in MK-2206-treated 2-cell embryos compared to their DMSO-treated counterparts. DNA was counterstained with DAPI. At least 20 embryos were used for each experiment. All experiments were repeated at least three times. Scale bars: 20 μm.

Next, we asked whether mouse embryos exhibit a similar regulation of H3K36me3. We treated zygotes (20 hphCG) with the inhibitors for 80 hr and found that inhibition of Akt or Pten blocked the majority of embryos at the 2-cell stage, whereas inhibition of Akt2 did not affect blastocyst formation [Fig. 7A]. We then treated the zygotes with Akt or Pten inhibitors and analyzed them with double immunostaining against H3 and H3K36me3. The experiment showed that inhibition of Akt reduced the level of H3K36me3 but not that of H3, whereas inhibition of Pten elevated the level of H3K36me3 with almost no effect on H3 level [Fig. 7B].

Next, we examined whether changes in H3K36me3 could affect Pol II CTD phosphorylation and whether simultaneous inhibition of Akt and Pten could recover the H3K36me3 level in treated embryos. Double immunostaining revealed that inhibition of Akt or Pi3k decreased the level of Ser2p, while inhibition of Pten slightly increased Ser2p [Fig. 7C], indicating that the changes in H3K36m3 reflected those in Pol II CTD Ser2p. Similarly, inhibition of Kdm4 elevated Ser2p. As we expected, simultaneous inhibition of Akt and Pten did not significantly alter H3K36me3 or Ser2p [Fig. 7C]. Interestingly, Akt2 inhibition had no clear effect on the levels of H3K36me3 or Ser2p. To ascertain whether the down-regulation of H3K36me3 and CTD Ser2p under Akt inhibition were due to reductions in the levels of Setd2 and Pol II, respectively, we examined these two factors in embryos subjected to Akt inhibition. Double immunostaining revealed that there was no clear change in the nuclear intensity of Pol II or Setd2 in MK-2206-treated embryos relative to control embryos [Fig. 7D]. However, PLA showed that the interaction of Setd2 with its target, H3K36me2, was seriously diminished and the fluorescence intensity of the PLA nuclear signal was dramatically lower in MK-2206-treated embryos compared to their control counterparts [Fig. 7E].

To examine whether Akt regulated H3K36me3 through Iws1, we evaluated the proximities of Akt and Iws1 in control and MK-2206-treated 2-cell (44 hphCG) embryos [Fig. 7F]. In siControl embryos, Akt was both nuclear and cytoplasmic but colocalized with Iws1 solely in nuclei. In the DMSO control group, we observed that Akt and Iws1 were close enough to possibly interact in nuclei. In MK-2206-treated embryos, in contrast, a barely detectable PLA signal was observed. These results indicate that in mouse early embryos, Iws1 regulates H3K36 trimethylation through Akt signaling.

We also tested the effect of Akt inhibition on mRNA splicing and mRNA nuclear export in our system. RT-qPCR analysis showed that the pre-mRNA splicing of *Cdk9* and *c-Myc* was defective in MK-2206-treated embryos [Fig. S5]. FISH revealed that Akt inhibition induced the accumulation of bulk mRNA in 2-cell embryos [Fig. 7G]. The fluorescence intensity of nuclear poly(A)+ mRNAs was significantly higher in MK-2206-treated embryos compared to the DMSO-treated group. Overall, our results collectively show that Iws1 is regulated by Akt to mediate the pre-mRNA splicing mark, H3K36me3, and mRNA export in mouse 2-cell embryos.

## Discussion

Two major gene expression events occur during mouse preimplantation development. First, two distinct bursts of transcription activate the embryonic genome mainly at the 2-cell stage. This event is required for normal development, as transcription inhibitors such as α-amanitin or flavopiridol block mouse embryos at the 2-cell stage^30,31^. Between the 4- and 8-cell stages, the second gene expression event activates cell fate specification genes, such as those encoding the transcription factors, *Nanog*, *Pou5f1*, and *Cdx2*^32^. Both events require that Pol II has access to gene promoters and transcription factors are available. Epigenetic marks play crucial roles in regulating Pol II promoter access and subsequent mRNA transcription, processing, and export. In addition to the epigenetic enzymes that write or erase these epigenetic marks, many nuclear factors have been shown to indirectly affect the architecture of the epigenome. Among them, Iws1 is known to form a complex with Supt6 to regulate H3K36 trimethylation and play crucial roles in mRNA processing and export. However, its role in embryogenesis has not yet been investigated. Here, we present the first description of the presence and expression patterns of Iws1 and Supt6 in mouse early development. Both factors are expressed in mouse preovulatory oocytes; their proteins are present and localized to the germinal vesicle, indicating that these factors are maternally provided and contribute to the early stages of development.

After fertilization, the mRNA expression levels of *Iws1* and *Supt6* peaked at the 2-cell stage. The nuclear intensities and proximities of the Iws1 and Supt6 proteins were also maximal at this stage, suggesting that their complex may play a role in EGA.

Embryos treated with *Iws1* siRNA or *Supt6* siRNA failed to form a compact morula, revealing that these factors are important in the early stages of development. There was no serious reduction of global transcription in KD embryos until the 8-cell stage, suggesting that this developmental failure might be due to defective post-transcriptional events.

We also observed that H3K36 trimethylation was dramatically decreased in *Iws1*-KD embryos. H3K36me3, but not -me2, is known to be regulated by Iws1 in both yeast and mammalian cells^20^, and Iws1 is reportedly required for the recruitment of Setd2 methyltransferase and the trimethylation of H3K36 in mammals^21^. Here, we observed that the nuclear intensity of Setd2 was not significantly altered in Iws1-KD embryos, but the proximities of Setd2 with H3K36me2 and Pol II CTD Ser2p were diminished. This confirms that Iws1 is crucial for the regulation of H3K36me3.

The developmental defect in KD embryos could not be directly ascribed to the decline in H3K36me3 levels, however, because we observed that *Setd2* depletion did not prevent blastocyst formation. Similarly, Zhang *et al*.^33^ reported normal blastocyst formation in *Setd2* KD mouse embryos.

Previous studies have revealed the importance of specific splicing factors in mouse early development. For example, SRp20, a splicing factor belonging to the highly conserved SR protein family, has been shown to be essential for preimplantation development^34^ and the splicing factors Sf3b1 and Sf3b14, which are members of the Sf3b multi-protein component of the U2 snRNP, are reportedly essential for mouse blastocyst formation^35^. Meanwhile, both Iws1 and Supt6 are known to be involved in the pre-mRNA splicing of mammalian cells. Yoh *et al*.^21^ showed that HeLa cells subjected to depletion of *SUPT6h* or *IWS1* exhibited Pol II-dependent transcripts with increased lengths and splicing defects. These transcripts remain mainly in the nucleus, indicating that defective splicing impairs mRNA export in these cells. The same study showed that an R1358K point mutation in the SUPT6h SH2 domain, which blocks the binding of SUPT6h to Pol II CTD Ser2p, yields the same phenotype. In addition, Sanidas *et al*.^29^ recently showed that human IWS1 plays a crucial role in pre-mRNA splicing by recruiting SETD2 to chromatin.

In embryos treated with si*Iws1* and/or si*Supt6*, we observed accumulation of bulk Poly(A)+ mRNA in the cell nuclei of blastomeres. This is in accordance with previous observations in human somatic cells^20,21^. Iws1 facilitates the interaction of the nuclear mRNA export factor, Alyref, with Pol II CTD. This interaction is crucial for the post-transcriptional mRNA export mediated by the TREX complex^36,37^, which interacts with exon-junction complex factors and is recruited to spliced transcripts^38^. Moreover, it has been shown that splicing promotes mRNA export^39^. Thus, we suggest that the developmental defects observed in *Iws1*- or *Supt6* KD embryos are likely to reflect defects in pre-mRNA splicing and/or mRNA export.

Liu *et al*.^24^ showed that human IWS1 is essential for cell viability and physically interacts with the arginine methyltransferase, PRMT5. Moreover, this interaction critically regulates the methylation of SUPT5h elongation factor and its interaction with Pol II. Indeed, IWS1 has been shown to physically interact with SUPT5h^24^. Since both Prmt5 and Supt5 are highly conserved in mouse and human, we hypothesized that a similar interaction exists in mouse early embryos. Indeed, our PLA experiments showed that Prmt5 resides in close proximity to and thus may interact with Iws1 in mouse early embryos, suggesting that a similar mechanism regulates Supt5 during mouse early development. This could explain the dramatic decline of transcriptional activity observed after the 8-cell stage in KD embryos. We also observed that Prmt5 potentially interacted with Nanog and that this interaction was decreased in si*Iws1* embryos. Studies have shown that Prmt5 is essential for mouse early development, and the pluripotency factors Nanog and Pou5f1 (Oct4) are downregulated in embryos lacking Prmt5^40^. Furthermore, Prmt5 and Nanog reportedly interact in mouse embryonic stem cells^27,41^, and a recent study found that Supt6 regulates Nanog and other pluripotency factors in mouse embryonic stem (ES) cells^42^. This suggests that the expression levels of pluripotency-related genes are regulated through a mechanism involving Iws1 and Supt6.

Given that the transcriptional activity and phosphorylation of Pol II CTD were decreased in DKD embryos, we speculate that Iws1 may also indirectly contribute to nascent RNA production by promoting Pol II CTD phosphorylation. As Iws1 plays a role in pre-mRNA splicing^29^, its downregulation could negatively affect the Ser2 phosphorylation of the CTD through the disruption of splicing. A recent study found that inhibition of general splicing by the specific small molecule inhibitors, SSA or PlaB (SF3B inhibitors), in human cells decreased the Ser2 phosphorylation of Pol II CTD^43^. The disruption of H3K36me3 reportedly has a negative effect on Pol II-mediated transcription elongation^44^. Iws1 is involved in exporting mRNA to the cytoplasm^20,21^ and disruption of mRNA export may negatively affect mRNA transcription^45^. Thus, the adverse effect of DKD on nascent RNA transcription observed in our experiments may reflect a decline in mRNA splicing and the subsequent decrease of Pol II CTD Ser2p, in addition to the DKD-associated inhibition of mRNA export. It can also be speculated that the nuclear retention of Poly(A)+ mRNA in DKD embryos was due not only to defective splicing, but also the poor interaction of mRNA export factors with Pol II CTD.

The effect of global splicing inhibition on mammalian early embryos has yet to be investigated. Several small molecules that specifically target Sf3b (e.g., SSA and PlaB) have been discovered in recent years^46^. To our best knowledge, however, their effect on mouse embryo development has not previously been reported. Here, we observed that SSA or PlaB blocked the majority of mouse embryos at the 2-cell stage [Fig. S6]. mRNA export was also severely impaired in these embryos, indicating that the above-described mechanism of splicing-dependent mRNA export is conserved in mouse early development.

The recruitment of SETD2 is regulated by the Akt3- and Akt1-mediated phosphorylation of IWS1 at Ser720/Thr721. SETD2 then trimethylates H3K36 to generate a docking site for the chromatin reader, MRG15, and the splicing regulator, PTB^29^. The Iws1 protein is highly conserved throughout evolution, and mouse Iws1 is 85% homologous to human IWS1. Therefore, it is logical to speculate that mouse Iws1 is also regulated by Akt-mediated phosphorylation. Akt signaling appears to be crucial for the abilities of mouse embryo cells and ES cells for both gaining and maintaining pluripotency^47–49^. All three variants of *Akt* are expressed during mouse early development^50^, and the Akt pathway is present and functional in preimplantation embryos^51^. Chemical inhibition of Pi3k^52^ or Akt^53^, or deletion of the master regulator genes, *Pdk1* or *Pten*^54^, all block mouse embryo development at the 2-cell stage. Here, we observed that Akt signaling also is required for H3K36 trimethylation in 2-cell mouse embryos. We were not able to confirm that Akt regulates K36me3 through phosphorylation of Iws1 due to the lack of a commercially available anti-phospho-Iws1 antibody. However, our PLA experiments revealed that Akt interacts with Iws1 in mouse early embryos and that Akt kinase activity is required for this interaction. We also found that Akt kinase activity was needed for proper pre-mRNA splicing of *Cdk9* or *c-Myc* [Fig. S5]. These observations reinforce the notion that, in mouse early embryos, Akt signaling regulates pre-mRNA splicing through the phosphorylation of Iws1.

After submission of our manuscript to this journal, a paper was published indicating the crucial role of Iws1 in mouse preimplantation embryo development^55^, supporting our findings in this study.

In conclusion, our study highlights the importance of the mRNA splicing and export factors, Iws1 and Supt6, in mouse embryonic genome activation and lineage-specific transcription factor regulation. Moreover, this study provides evidence indicating that the function of Iws1:Supt6 is regulated by the Akt signaling pathway during mouse early embryogenesis.

## Materials and Methods

### Cell culture and preparation of *in vivo*-derived mouse oocytes and embryos

All animal care and use procedures were approved by the Institutional Animal Care and Use Committee of Chungnam National University and all methods were performed in accordance with the relevant guidelines and regulations. NIH/3T3 cells were cultured on coverslips for 24 hrs in 5% CO_2_ at 37 °C in DMEM supplemented with 10% fetal bovine serum. Cells were then subjected to experiments. Oocytes and embryos were obtained from B6D2 F1 female mice (Charles River). Superovulation was induced in three- to five-week-old females by injection with 7.5 IU PMSG (Sigma). Forty-four hr later, the females were injected with 5 IU hCG and coupled with males. Zygotes and embryos were collected into FHM HEPES buffered medium (MR-025-D, Specialty Media, Millipore) and cultured in KSOMaa (MR-121-D, Specialty Media, Millipore).

### Antibodies

The following antibodies were used in our immunostaining or proximity ligation assays: rabbit antibodies against Iws1 (5681S, Cell Signaling), H3K36me3 (61101), Pol II CTD Ser2p (91116, both from Active Motif), Setd2 (ab69836), H3K36me2 (ab9049), H3K36me1 (ab9048), Cdx2 (ab76541) and Nanog (ab109250, all from Abcam); and mouse antibodies against Supt6 (sc-393920), Pol II N-terminus (sc-55492), Alyref (sc-32311), Pou5f1 (sc-5279), Nanog (sc374103), Prmt5 (sc-376937), Cyclin T1 (sc-271575), Cyclin H (sc-1662); and rabbit antibodies against Cdk9 (sc-484) and Cdk7 (sc-529, all from Santa Cruz), H3K36me2 (61019) and Histone H3 (39763, both from Active Motif), Pol II CTD Ser2p (MMN-129R) and Pol II CTD Ser5p (MMH-134R, both from Covance), SC35 (S4045, Sigma), and Akt (2920S, Cell Signaling).

### Iws1, Supt6, and Setd2 siRNA and electroporation

The following siRNAs were designed in-house: *Iws1* siRNA (si*Iws1*#1) 5′ GCCGAGCAGUGAUGUAUCUGUdTdT 3′; *Supt6* siRNA (si*Supt6*#1) 5′ GUGUGGCAGUGGGAUGAGAAdTdT 3′; *Setd2* siRNA (si*Setd2*#1) 5′ GGAAUUGCUCUCGUUUCAUGAdTdT 3′. The following siRNA were obtained from Sigma: Mouse *Iws1* MISSION esiRNA (si*Iws1*#2, EMU022811); Mouse *Supt6* MISSION esiRNA (si*Supt6*#2, EMU046481); and Mouse *Setd2* MISSION esiRNA (si*Setd2*#2, EMU214881). Each siRNA was diluted in Opti-MEM 1 (Invitrogen) to a final concentration of 40 µg/ml, put into a 4-mm cuvette (Gene Pulser 165–2088, Bio-Rad) and connected via a cuvette chamber (ShockPod, Bio-Rad) to a Gene Pulser Xcell electroporator (Bio-Rad). The zygotes were plunged into the mixture, exposed to three pulses (30 V, 5 msec each; interval, 1 sec), immediately removed from the cuvette and washed three times in KSOMaa culture medium. Then, zygotes (n = ~25 per group) were placed in 50-µl droplets of medium and covered with mineral oil until further analyses.

### Treatment with chemical inhibitors

The following small molecule inhibitors and their final concentrations were used in this study: MK-2206 (S1078, 10 μM), CCT128930 (S2635, 500 nM), both from SelleckChem, LY-294002 (L9908, 10 μM), VO-OHpic (V8639, 1 μM) and ML-324 (SML0741, 15 μM) all from Sigma. The inhibitors were dissolved in DMSO and applied at the indicated final concentrations. Spliceostatin A (200 nM), dissolved in methanol, was a kind gift from Dr. Minoru Yoshida (Chemical Genetics Laboratory, RIKEN, Japan), and Pladienolide B (CAS 445493-23-2, (100 nM)) was purchased from Santa Cruz and was dissolved in DMSO and were applied at the indicated final concentrations.

### Fluorescence *in situ* hybridization (FISH)

After fixation in 2% formaldehyde for 15 minutes, oocytes or embryos were washed twice in PBS/PVA for 5 min at room temperature. Then the specimens were permeabilized with 1% Triton X-100 in PBS for 30 min followed by two washes in saline-sodium citrate (SSC) plus 50% formamide for 5 min at room temperature. Hybridizations were performed overnight at 37 °C in a hybridization buffer consisting of 10% dextran sulfate, 2 mM vanadyl ribonucleoside complex, 0.02% BSA, 40 μg tRNA and 10 ng of RNA probe in 2x SSC plus 50% formamide. Thereafter, the samples were washed with pre-warmed 2x SSC/50% formamide at 37 °C for 30 min, washed twice in PBS/PVA and mounted in Vectashield mounting medium containing DAPI (Vector Laboratories). The probes corresponding to mouse *Iws1* (FAM-5′ CCCCCGTCACGTCCTTCCTGGCCGAGTGACCTCTCTCC 3′), mouse *Supt6* (Cy3-5′ GCCGCTGTTTCTGTTTCCGTCGTTCTGCCTCCTTTT 3′), Cy3-5′ oligo-dT (30-mer) and their complementary sense probes were produced by Bioneer, Korea.

### Immunofluorescence staining

After two washes in 0.1% (w/v) PVA in PBS, the specimens were fixed at room temperature in 2% (v/v) formaldehyde in PBS for 15 min. Then, the samples were permeabilized in PBS containing 0.5% (v/v) Triton X-100 for 30 min and washed for 10 min in 100 mM glycine in PBS to inactivate free aldehyde groups. The specimens were then exposed to 3% (w/v) BSA for 20 min followed by 5 min in PBS containing 0.5% (w/v) BSA and 0.1% (w/v) gelatin from the skin of cold-water fish (PBG, Sigma) for blocking nonspecific binding sites. Samples were incubated with primary antibodies in PBG for 16 hrs at 4 °C followed by four washes PBG for 5 min each time, and then incubated with the appropriate secondary antibodies for 1 hr in PBG at room temperature. The samples were then washed twice in PBG and twice in PBS (5 min per wash), and finally deposited on slides and mounted under coverslips using Vectashield mounting medium containing DAPI for microscopic observation.

### 5-Ethynyluridine (EU) incorporation assay

Nascent RNA transcripts were detected using a Click-iT RNA imaging kit (C10330, Invitrogen). Briefly, embryos were incubated in 1 mM EU in KSOMaa for 1 hour in 37 °C, washed in PBS/PVA, fixed in 2% formaldehyde in PBS/PVA for 5 min, permeabilized in 1% Triton X-100 plus 100 mM glycine in PBS for 30 min and labeled according to the manufacturer’s protocol. The labeled embryos were mounted in Vectashield with DAPI.

### The proximity ligation assay (PLA)

PLA was performed using a Duolink *In Situ* Red Starter Kit Mouse/Rabbit (DUO92101, Sigma). Oocytes or embryos were fixed and processed exactly as described for our immunofluorescence staining, and the PLA probes were visualized as previously described [Panamarova *et al*., 2016]. Briefly, the embryos were washed three times with PBS containing 0.05% Tween 20, incubated with the PLA PLUS and MINUS probes (a 1:5 dilution in antibody diluent solution) for 1 hr at 37 °C, and washed three times (5 min each) in washing buffer A (0.01 M Tris, 0.15 M NaCl, and 0.05% Tween 20 in high purity water). To ligate and circularize the two DNA oligonucleotides, embryos were incubated in a ligation-ligase solution (ligation mix 1:5, ligase 1:40, in high purity water) for 30 min at 37 °C. The embryos were washed three times in washing buffer A, incubated in the amplification and polymerase mixture (amplification mix 1:5, polymerase 1:8, in high purity water) for 100 min at 37 °C, washed three times (10 min each) in washing buffer B (0.2 M Tris and 0.1 M NaCl in high purity water), and then mounted on slides with Vectashield containing DAPI. The PLA signals were visualized by confocal laser-scanning microscopy (LSM 710 META; Zeiss, Jena, Germany).

### RNA extraction and RT-qPCR analysis

Oocytes or embryos were stored at −80 °C until analysis. For gene expression assays, total RNA was extracted from each sample (n = 50) using an RNeasy Mini Kit (Cat. No. 74104; Qiagen, Valencia, CA, USA) and an RNase-Free DNase Set (Cat. No. 79254; Qiagen), and cDNA was prepared with a TOPscript™ RT DryMIX kit (Enzynomics, Daejeon, Republic of Korea) in accordance with the manufacturer’s instructions. RT-qPCR was conducted with a TOPreal™ qPCR 2X PreMIX (SYBR Green with low ROX) kit (Enzynomics) on a CFX96 Touch Real-Time PCR Detection System (Bio-Rad). PCRs with no template controls were performed for each primer pair. Relative mRNA expression levels were analyzed using the 2−ΔΔCt method. *H2afz* was used as an internal standard. The primer sequences are listed in Table S1.

For splicing assays, 100 embryos per group were treated with 200 μM of 5-ethynyl-uridine for 1 hr. Total RNA was extracted from the treated embryos using TRIzol (Life Technologies), purified using a Click-iT Nascent RNA Capture Kit (Life Technologies) and biotinylated by the click reaction. Biotinylated RNA was purified using streptavidin beads. cDNA was synthesized using a SuperScript VILO cDNA Synthesis Kit (Life Technologies) and RT-qPCR and relative quantification analyses were performed with an MX3000P system (Agilent, Santa Clara, CA, USA) using SYBR Green dye chemistry. The *18 S* rRNA was used as an internal control.

### Confocal microscopy and fluorescence intensity measurement

A Zeiss scanning laser confocal microscope running the Zeiss LSM Image Browser software was used for image acquisition. Serial optical (Z-series) sections with 0.5-μm intervals were captured and the staining patterns and intensities of all nuclear areas were imaged. For each fluorescence intensity measurement, all oocyte and embryo samples were prepared and processed simultaneously. The laser power was adjusted to ensure that the signal intensity was below saturation for the specimen that displayed the highest intensity, and all images were next scanned at that laser power. For quantification, fluorescence signals were normalized to DAPI. ImageJ measure function was used for intensity measurement in normalized sections.

### Statistical analysis

All data were subjected to one-way analysis of variance (ANOVA) followed by the Fisher’s protected least significant difference (LSD) test. Analyses were applied using the SPSS software. At least three replicates were performed for each experiment. A *P* value less than 0.05 denoted a statistically significant difference and indicated by * for *p* < 0.05, ** for *p* < 0.01 and *** for *p* < 0.005.

## Supplementary information

supplementary information
