# Supplementary material for: Iws1 and Spt6 Regulate Trimethylation of Histone H3 on Lysine 36 through Akt Signaling and are Essential for Mouse Embryonic Genome Activation
Source: Sci Rep. 2019 Mar 7;9:3831. doi: 10.1038/s41598-019-40358-3 (PMC6405902; doi:10.1038/s41598-019-40358-3)
Supplement: Supplementary file 1 — supplementary information [file 41598_2019_40358_MOESM1_ESM.pdf]

Iws1 and Spt6 Regulate Trimethylation of Histone H3 on Lysine 36 through Akt Signaling and are Essential for Mouse Embryonic Genome Activation

Reza K. Oqani, Tao Lin, Jae Eun Lee, Jeong Won Kang, Hyun Young Shin, Dong Il Jin.

Department of Animal Science & Biotechnology, Research Center for Transgenic Cloned Pigs, Chungnam National University, Daejeon 34134, Republic of Korea

*Corresponding author: Dong Il Jin. Tel.: +82 42 821 5876. E-mail: dijin@cnu.ac.kr

Table S1. The RT-qPCR primers used in this study

Name	Primer sequence
Iws1	Forward: ATTGCATTTCTCTGAGGGCCGTGGGTA<br>Reverse: GCTGTAGTACTCCGAGTCCATGGCA
Supt6:	Forward: TTAACGCAGCAGCGGCGACGGT<br>Reverse: GCTTGAAGGCCGCTGCTTCTCCGCTA
Pou5f1	Forward: ATGGGGAAAGAAGCTCAGTG<br>Reverse: CAAAATGATGAGTGACAGACAGG
Nanog	Forward: TTCTTGCTTACAAGGGTCTGC<br>Reverse: AGAGGAAGGGCGAGGAGA
Cdx2	Forward: AGCTGCTGTAGGCGGAATGTATG<br>Reverse: TCAGTGACTCGAACAGCAGCAA
H2afz	Forward: TCCAGTGGACTGTATCTCTGTGA<br>Reverse: GACTCGAATGCAGAAATTTGG
18S rRNA	Forward: ATGCGTGCATTTATCAGATCAAAACC<br>Reverse: AAAGTGGACTCATTCCAATTACAGG
Cdk9 Exon 1	Forward: TGGAGGTGGCCATGGCCAAGCAG<br>Reverse: AATGTGCCTTGGCCGATCTTGGCAA
Cdk9 Exon 2	Forward: AAGTCTTTAAAGCCAAGCACCGTCAG<br>Reverse: CCCTCCTTCTCATTCTCCATCAA
Cdk9 Exon 1-2, spliced	Forward: AAGTACGAGAA ACTTGCCAAGATCG<br>Reverse: CTGACGGTGCTTGGCTTTAAAGAC
Cdk9 Exon 1-2, unspliced	Forward: AAGTACGAGAAACTTGCCAAGATCG<br>Reverse: GGTTAGAAGAAGGTCGGCCCAGTGG
c-Myc Exon 1	Forward: AACTTTGCCCATTGCAGCGGGCAGACA<br>Reverse: GGAAATCCAGCCTTCAAACAGCTCG
c-Myc Exon 2	Forward: TATGACCTCGACTACGACTCCGTACA<br>Reverse: GGAGAAGGACGTAGCGACCGCAACATA
c-Myc Exon 1-2, spliced	Forward: CCTCGAGCTGTTTGAAG GCTGGATTTCC<br>Reverse: AGTTCACGTTGAGGGGCATCGTCGT
c-Myc Exon 1-2, unspliced	Forward: CCTCGAGCTGTTTGAAG GCTGGATTTCC<br>Reverse: CGCAGAAAGAACACAGGGAAAGACCACC

Figure S1. Effect of *Setd2* siRNA on H3K36me1, H3K36me2, and H3K36me3 signals. Mouse zygotes were subjected to si*Setd2* or siControl and then were cultured until the blastocyst stage. Only H3K36me3 was reduced in si*Setd2* embryos. More than 50 embryos were analyzed in each group. Scale bar: 20 μm.

Figure S2. Dynamic mono- and dimethylation of H3K36 on lysine36 in mouse preimplantation embryos. Shortly after fertilization, strong staining of H3K36me1 is associated with the chromatin of both newly formed pronuclei, whereas only the smaller pronucleus closer to the polar body (i.e., the female pronucleus) exhibits H3K36me2 staining. At the 2-cell stage, H3K36me3 remains restricted to half (i.e., the maternal half) of the genome. After the 2-cell stage, both modifications are seen on the chromatin of all analyzed stages. In blastocysts, H3K36me1 may be seen throughout the chromatin of the entire embryo, whereas H3K36me2 is mainly detected in ICM cells. Scale bar: 20 μm.

Figure S3. Pol II CTD kinase nuclear signals in si*Iws1*;*Supt6* 2-cell embryos. Double-immunostaining of Cdk9/Cyclin T1 and Cdk7/Cyclin H reveals that the nuclear signals are unchanged in DKD embryos compared to control embryos. Scale bar: 20 μm.

Figure S4. H3K36me3 is regulated through Pi3k/Akt pathway in mouse fibroblasts. Treatment with an Akt inhibitor (MK-2206) or a Pi3k inhibitor (LY-294002) decreases the level of global H3K36me3 without altering the level of H3 in mouse 3T3 cells. Treatment with a

Pten inhibitor (VO-OHpic) or a Kdm4 inhibitor (ML-324) increases the level of H3K36me3. The graph depicts the mean values of signal intensities. Scale bar: 10 μm.

Figure S5. Effect of Akt inhibition on pre-mRNA splicing in 2-cell embryos. The pre-mRNA splicing of *Cdk9* and *c-Myc* is defective in MK-2206-treated embryos whereas treatment with the Akt2 inhibitor, CCT128930, does not affect this splicing. The levels of spliced and unspliced intron 1 were quantified by RT-qPCR. The expression level of the 18S rRNA in siControl cells was set as 1 and used as an internal control.

Figure S6. Effect of global splicing inhibition on mouse embryo development, H3K36me3 level, and Pol II CTD phosphorylation. (A) Treatment with the splicing inhibitors, Spliceostatin A (SSA) or Pladienolide B (PlaB), blocks embryonic development at the 2-cell stage. **(B)** SSA (200 nM) or PlaB (100 nM) significantly reduce the levels of Pol II CTD Ser2 phosphorylation and H3K36me3. **(C)** The graph depicts the fluorescence intensity of nuclear signals corresponding to Pol II CTD Ser2 phosphorylation and H3K36me3. **(D)** SSA or PlaB negatively affect CTD Ser5 phosphorylation (CTD S5p) but have little effect on the pan Pol II nuclear signal. Scale bar: 20 μm.

Figure S1

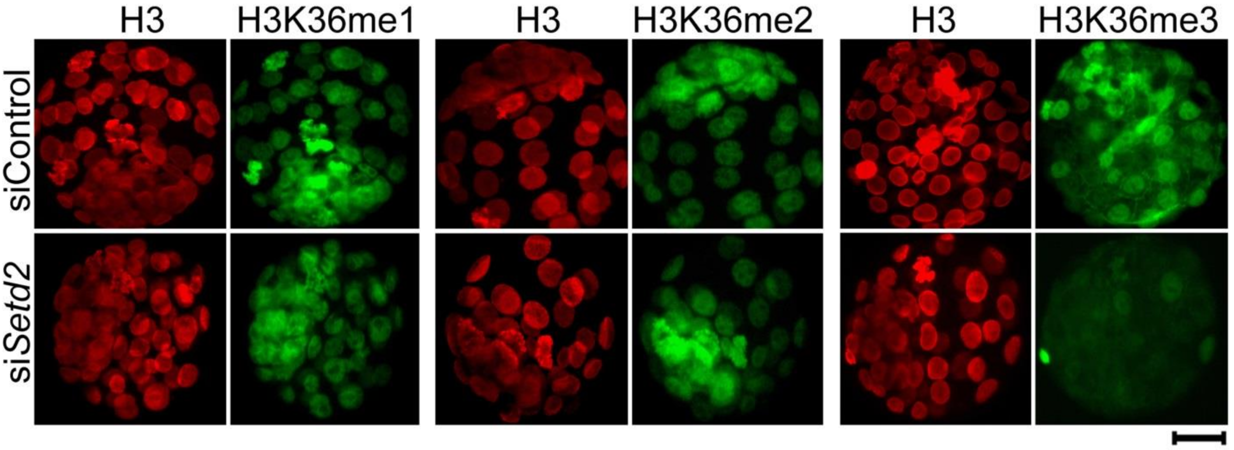

Figure S2

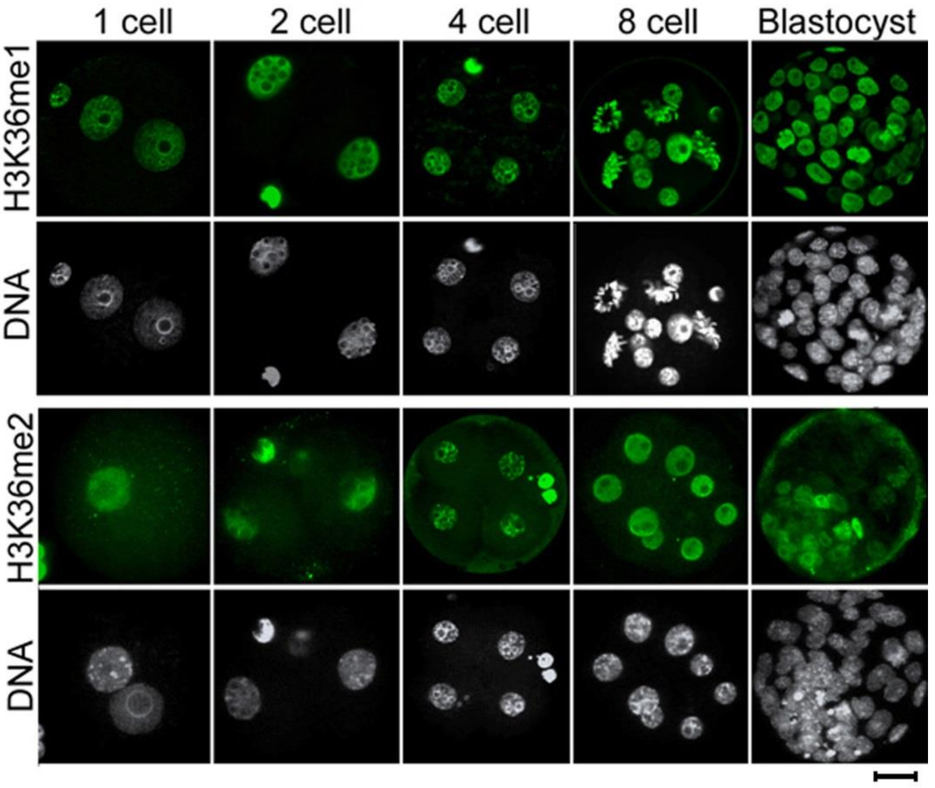
1 cell
2 cell
4 cell
8 cell
Blastocyst
H3K36me1
DNA
H3K36me2
DNA

Figure S3

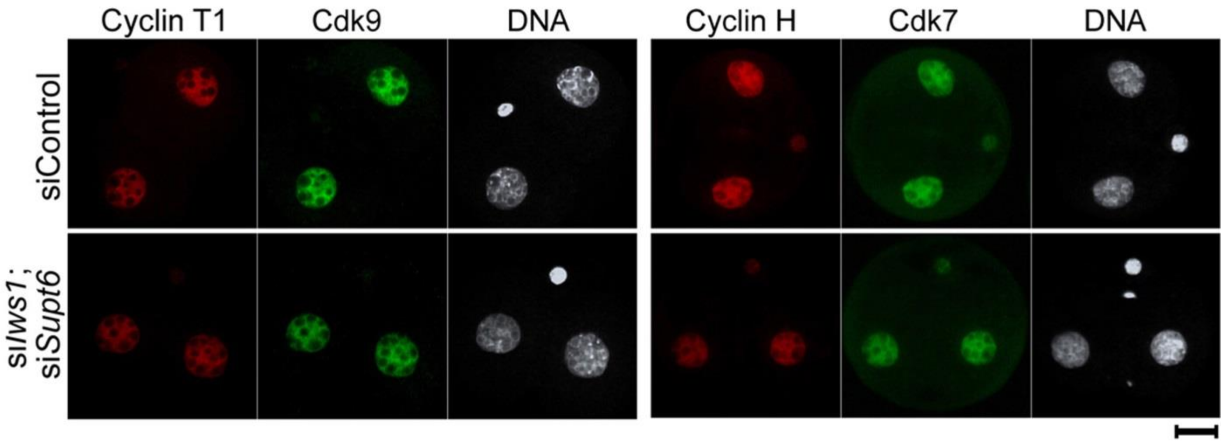
Cyclin T1
Cdk9
DNA
Cyclin H
Cdk7
DNA
siControl
siIws1; siSupt6

Figure S4

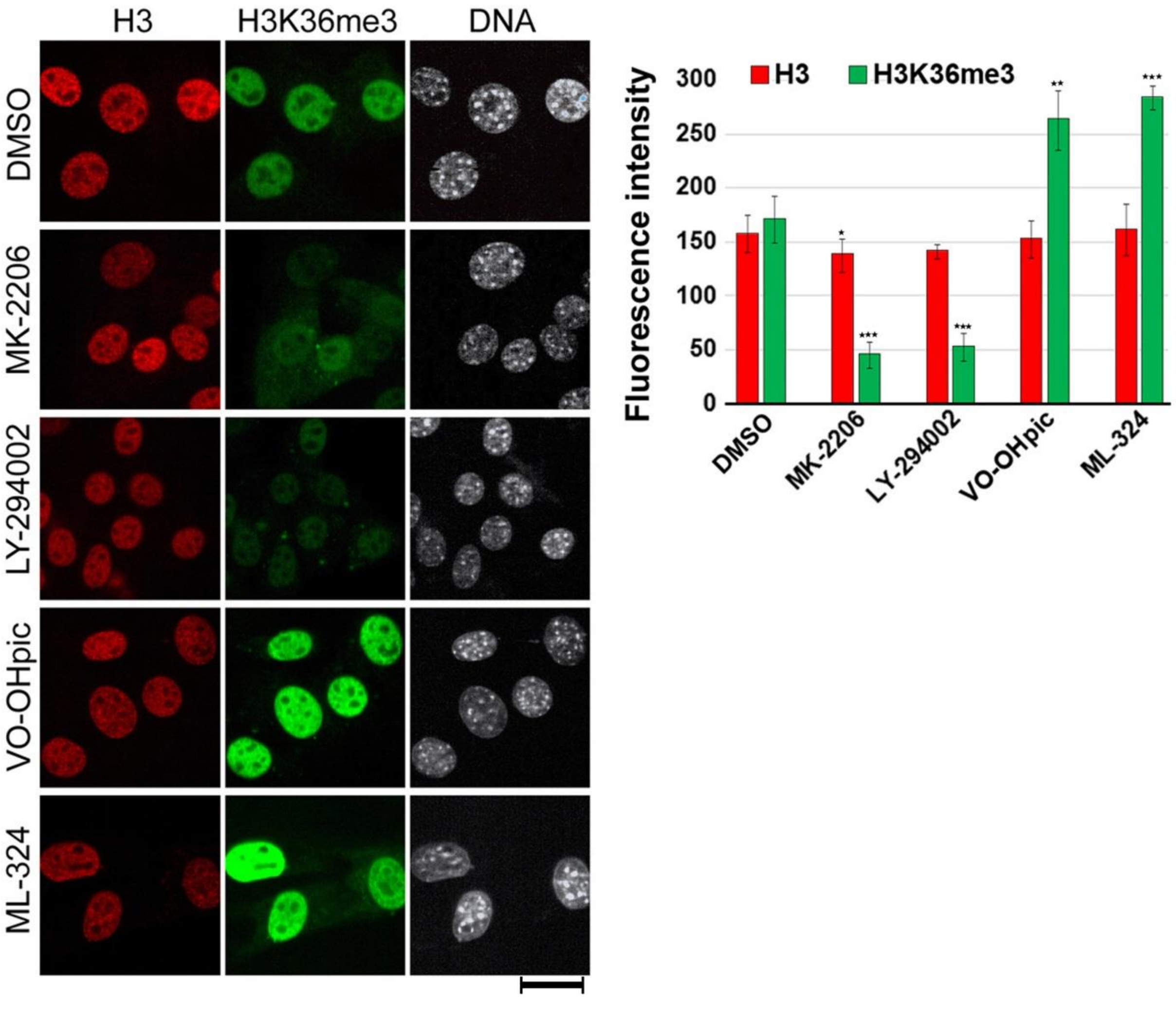
H3
H3K36me3
DNA
DMSO
MK-2206
LY-294002
VO-OHpic
ML-324
H3
H3K36me3
Fluorescence intensity
300
250
200
150
100
50
0
*
***
***
**
***
DMSO
MK-2206
LY-294002
VO-OHpic
ML-324

Figure S5

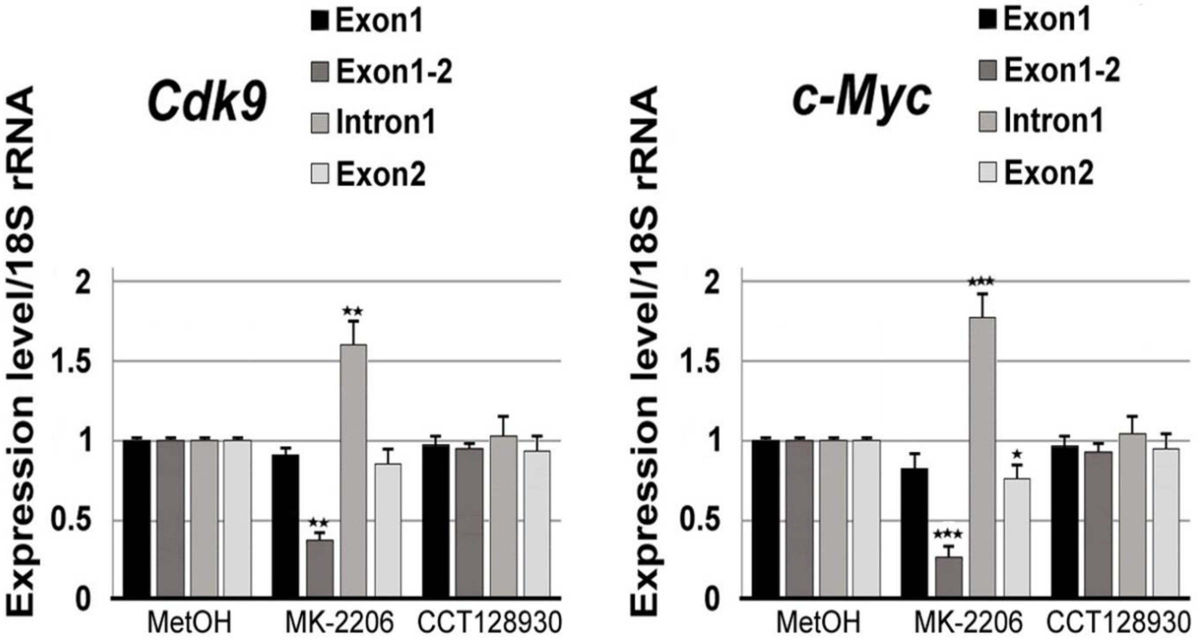
Cdk9
Exon1
Exon1-2
Intron1
Exon2
Expression level/18S rRNA
2
1.5
1
0.5
0
MetOH
MK-2206
CCT128930
c-Myc
Exon1
Exon1-2
Intron1
Exon2
Expression level/18S rRNA
MetOH
MK-2206
CCT128930

Figure S6

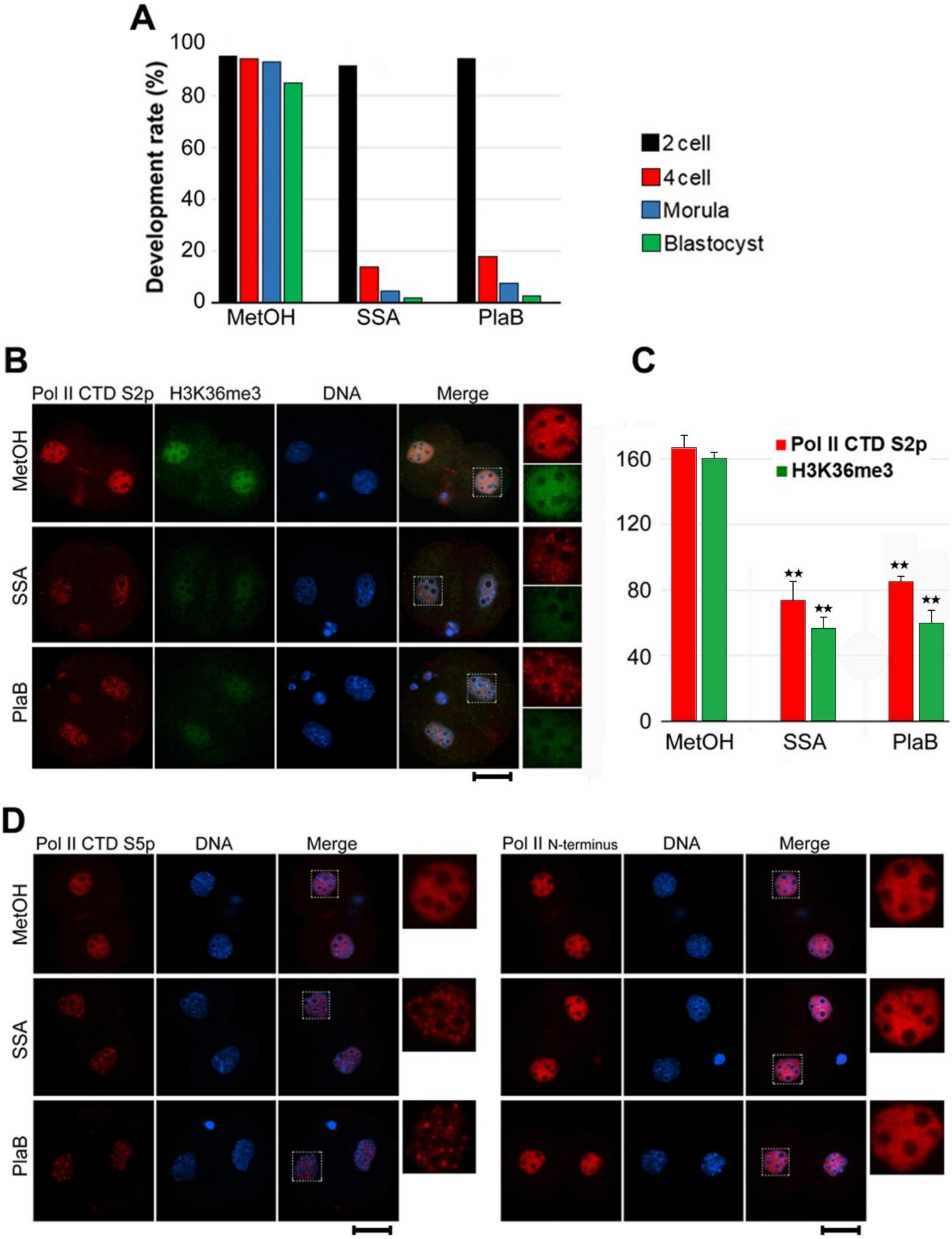
A
Development rate (%)
100
80
60
40
20
0
MetOH
SSA
PlaB
2 cell
4 cell
Morula
Blastocyst
B
Pol II CTD S2p
H3K36me3
DNA
Merge
MetOH
SSA
PlaB
C
Pol II CTD S2p
H3K36me3
160
120
80
40
0
MetOH
SSA
PlaB
D
Pol II CTD S5p
DNA
Merge
Pol II N-terminus
DNA
Merge
MetOH
SSA
PlaB
